# Anti-inflammatory and anti-infectious effects of *Evodia rutaecarpa *(*Wuzhuyu*) and its major bioactive components

**DOI:** 10.1186/1749-8546-6-6

**Published:** 2011-02-14

**Authors:** Jyh-Fei Liao, Wen-Fei Chiou, Yuh-Chiang Shen, Guei-Jane Wang, Chieh-Fu Chen

**Affiliations:** 1 Institute of Pharmacology, National Yang-Ming University, No 155, Sec 2, Linong Road, Taipei 112, Taiwan; 2 National Research Institute of Chinese Medicine, No 155-1, Sec 2, Linong Road, Taipei 112, Taiwan

## Abstract

This article reviews the anti-inflammatory relative and anti-infectious effects of *Evodia rutaecarpa *and its major bioactive components and the involvement of the nitric oxide synthases, cyclooxygenase, NADPH oxidase, nuclear factor kappa B, hypoxia-inducible factor 1 alpha, reactive oxygen species, prostaglandins, tumor necrosis factor, LIGHT, amyloid protein and orexigenic neuropeptides. Their potential applications for the treatment of endotoxaemia, obesity, diabetes, Alzheimer's disease and their uses as cardiovascular and gastrointestinal protective agents, analgesics, anti-oxidant, anti-atherosclerosis agents, dermatological agents and anti-infectious agents are highlighted. Stimulation of calcitonin gene-related peptide release may partially explain the analgesic, cardiovascular and gastrointestinal protective, anti-obese activities of *Evodia rutaecarpa *and its major bioactive components.

## Introduction

Inflammation is a protective physiological response of an organism to chemical, physical, infectious agents, environmental toxins, ischemia or an antigen-antibody interaction. However, prolonged or overactive inflammation may cause tissue damage. Inflammation is very common manifested as body temperature change, edema, itch and pain, occasionally as serious as septic shock, tissue cirrhosis, necrosis or cancer. In the United States, over 500,000 patients suffer from sepsis triggered by severe systemic inflammation per year [1].

Various factors are involved in inflammation, such as calcium homeostasis, histamine, bradykinin, serotonin (5-HT), eicosanoids (prostaglandins, PG; thromboxanes, TX; leukotrienes, LT), platelet-activating factor, hormones (corticosterones), cytokines, interleukins (IL), chemotaxics, cyclooxygenase (COX), adhesion molecules, reactive oxygen species (ROS) (H_2_O_2_, O_2_^-^), nitric oxide (NO) and substance P. Cells taking part in inflammation are erythrocytes, neutrophils, basophils, eosinophils, platelet, natural killer cells, lymphocytes, mast cells, antigen presenting cells and dendritic cells [2]. Diseases and syndromes, such as arthritis, atherosclerosis, atopic dermatitis, brain or heart stroke, cancer, cataract, diabetes, neurodegeneration, pain, rhinitis and septic shock, are all related to inflammation.

Natural products may still be the most abundant sources for new drug development. Aspirin and corticosterone are two well known examples for anti-inflammatory products derived from Nature. Favonoids are potential therapeutic agents for the treatment of inflammation, heart disease and cancer [3]. This article reviews the anti-inflammatory relative and anti-infectious effects of *Evodia rutaecarpa *(*Wuzhuyu*) and its major bioactive components such as dehydroevodiamine (DeHE), evodiamine (Evo) and rutaecarpine (Rut).

Mechanisms of anti-inflammatory relative effects of *Evodia rutaecarpa *and its bioactive components are summarized in Additional file 1.

### Effects on nitric oxide (NO) system and nitric oxide synthase (NOS)

While NO is involved in the blood pressure regulation, smooth muscle relaxation, platelet aggregation, neurotransmission, long-term potentiation, penile erection, apoptosis and immune response, over-expression of inducible nitric oxide synthase (iNOS) plays an important role in systemic or local, acute or chronic inflammation such as septic shock and rheumatoid arthritis [4,5].

A study on the cardiovascular effects of DeHE, Evo and Rut found that Rut produced a full NO-dependent vasodilatation whereas Evo and DeHE produced a partially endothelium-dependent effect at 50% and 10% respectively [6]. Apart from endothelium dependence, alpha 1-adrenoceptor blockade, K^+ ^channel activation and Ca^2+ ^channel blockade were also involved in the vasorelaxant effect of DeHE [7]. Coupled with influx of extracellular calcium, Rut produced the endothelium-dependent vasorelaxant effect by activation of endothelium NOS and release of NO without pertussis toxin-sensitive Gi protein and other G proteins or phospholipase C activation being involved [8]. Another study using the whole-cell patch-clamp method found that Rut inhibited the L-type voltage-dependent calcium channels of rat vascular smooth muscle cells and increased NO release through opening of non-voltage-dependent calcium channels in the endothelial cells [9,10].

In other smooth muscles, Evo was shown to possess a potent corporal relaxing effect attributed to endothelium-independent properties and was tested as a potential agent for the treatment of erectile dysfunction in aged animals [11].

DeHE was found to inhibit NO production by interfering not only with the priming signal initiated by interferon-gamma but also with iNOS synthesis while Evo affected the former only [12]. Ethanol extract of *Evodia rutaecarpa *dose-dependently prevented the circulation failure, vascular hyporeactivity to phenylephrine, liver dysfunction and reduced the NO over-production in plasma in lipopolysaccharide (LPS)-induced endotoxaemic rats [13]. *Evodia rutaecarpa *ethanol extract exhibited potent antioxidative effects in neutrophils and that in microglial cells *Evodia rutaecarpa *ethanol extract, DeHE, Evo and Rut all inhibited the LPS-induced NO production and iNOS expression [14].

### Effects on nuclear factor kappa B (NF-kappa B), cyclooxygenase (COX), 5-lipoxygenase(5-LO), prostaglandins (PG), serotonin (5-HT), interleukins (IL), tumor necrosis factor-alpha (TNF-α) and LIGHT

COX and LO are enzynes involved in the metabolism arachidonic acid, thus formation of PG, IL, and other metabolites which related to inflammation [2].

A study found that Evo and Rut strongly inhibited PGE_2 _synthesis in LPS-treated RAW 264.7 cells and that Evo but not Rut inhibited COX-2 induction and NF-kappa B activation. Goshuyuamide ||, another *Evodia rutaecarpa *active component, inhibited 5-LO, thereby reducing leukotriene (LT) synthesis; however, these three compounds did not inhibit iNOS mediated NO production from cells up to 50 μM [15]. Another study reported that DeHE inhibited LPS-induced iNOS and COX-2 and their mRNAs expression in RAW 264.7 cells, probably through the suppression of NF-kappa B activation in the transcriptional level [16]. Evo was found to inhibit hypoxia-induced inflammatory response by repressing not only COX-2, COX-2 mRNA and iNOS expression but also PGE_2 _release in a concentration-dependent manner in RAW264.7 cells under hypoxia condition, mediated *via *dephosphorylation of the serine/threonine kinases Akt and p70S6 kinase regulating the translation process of hypoxia-inducible factor-1 alpha by Evo [17]. A study demonstrated that Rut is a new class of COX-2 inhibitor partially contributing its *in vivo *anti-inflammatory activities on lamda-carrageenan induced paw edema in rats [18].

Wuzhuyu Tang (WT), a Chinese medicine formula for migraine treatment, is composed of Evodia fruit, Ginger, Ginseng, and Jujube. A study on WT reported regulatory effects of various components in WT on tryptophan hydroxylase 2 (TPH2, the rate limiting enzyme for 5-HT biosynthesis in brain) promoter, suggesting that the effects of WT on migraine could be due to its stimulating effects on TPH2 promoter and promotion of the 5-HT synthesis and release in the brain [19].

In human mononuclear cells, 10% to 30% of *Evodia rutaecarpa *extracts were found to stimulate the secretion of IL-1 beta, IL-6, TNF-α and granulocyte-macrophage colony-stimulating factor; however, more than 40% of *Evodia rutaecarpa *extract lost its stimulating effect. *Evodia rutaecarpa *extract showed better stimulating effect when reacted with mononuclear cell for 18 or 24 hours than one or three hours [20,21].

Homologous to Lymphotoxin, exhibits inducible expression, competes with Herpes Simplex Virus Glycoprotein D for binding to Herpes Virus entry Mediator (HVEM), a receptor on T lymphocytes (LIGHT) showed inducible expression and acted as a new player in the atherogenesis [22]. Evo and Rut decreased LIGHT-induced production of ROS, IL-8, monocyte chemoattractant protein-1, TNF-α, IL-6, and the expression of chemokine receptor (CCR) 1, CCR2 and intracellular adhesion molecule 1 and the phosphorylation of extracellular-signal-regulated kinases (ERK) 1/2 and p38 mitogen-activated protein kinase (MAPK) *via *decreasing ROS production and NADPH oxidase activation. Evo and Rut were considered as potential anti-atherosclerosis agents [23].

### Capsaicin-like effects

Used as an analgesic, capsaicin, the major bioactive component of *Capsicum frutescens *L., is a vanilloid receptor agonist [24]. Capsaicin-sensitive sensory neurons are nociceptive neurons that release calcitonin gene-related peptide (CGRP) on activation. Capsaicin-sensitive sensory neurons are rich in transient receptor potential channel vanilloid type 1 (TRPV1) which plays a fundamental role in pain and involves in the protective effects on cardiovascular and gastrointestinal systems. A study found that TRPV1 could be activated by endogenous cannabinoids (anandamide, N-archidonoyl dopamine, N-oleoyldopamine) or by exogenous agonists such as capsaicin, Evo and Rut which in turn stimulated the CGRP relaease [25].

An earlier study found that oral administration of ethanol extract of *Evodia rutaecarpa *to mice reduced the acetic acid induced abdominal stretch [26]. Another study confirmed that Evo and Rut were partially responsible for the analgesic effects [27]. Limonin from *Evodia rutaecarpa *was also found to be analgesic [28].

Evo possesses vanilloid receptor agonistic activities comparable to capsaicin in guinea-pig isolated bronchus [29] and atria [30], and suppresses acetic acid-induced writhing by desensitizing visceral sensory nerves [31]. A study found that Evo was an agonist for the vanilloid receptor TRPV1 in rat, about 3-19 fold less potent than capsaicin [32]. Moreover, Evo was found to protect bovine serum albumin induced guinea-pig cardiac anaphylaxis by stimulation of CGRP release [33] and exert protection against myocardial ischemia-reperfusion injury in rats by activation of vanilloid receptors to stimulate the CGRP release [34].

Rut did not demonstrate bronchoconstrictive effects in guinea-pig isolated bronchus [29]. Rut increased the CGRP and decreased TNF-α with significant improvement of cardiac function and inhibition of the sinus tachycardia in antigen induced cardiac anaphylactic injury of guinea-pig hearts [35]. Rut was also found to release CGRP to inhibit vasoconstriction induced by anaphylaxis in guinea-pigs [36]. Similarly, the cardio-protective effect of Rut on myocardial ischemia-reperfusion injury was caused by vanilloid receptor activation to evoke CGRP release in normal [37] or spontaneously hypertensive rats (SHR) [38]. Rut inhibited hypoxia/reoxygenation induced apoptosis in primary rat hippocampal neurons *via *TRPV1-(Ca^2+^)_i_-dependent and phosphoinositide 3-kinase (PI3K)/Akt signaling pathway [39]. Furthermore, the protective effects of Rut on acetylsalicylic acid and stress-induced gastric mucosa injury were related to stimulation of endogenous CGRP release *via *activation of vanilloid receptor [40]. Rut also protected the gastric mucosa against injury induced by ethanol *via *stimulating the release of CGRP to attenuate ethanol-induced elevation of asymmetric dimethylarginine levels [41].

A review article reported that CGRP played an important role in the initiation, progression and maintenance of hypertension and that in contrast the increase in CGRP levels or the enhancement of vascular sensitivity response to CGRP served as a beneficial compensatory depressor role in the development of hypertension [42]. Furthermore, there are therapeutic possibilities of CGRP in hypertension [43]. Effects of Rut on cardiovascular system were reported to act through the release of CGRP, including the depressor and vasodilator [44], the hypotensive effects in the phenol-induced hypertensive rats [45], the hypotensive effects and reduction of mesenteric artery hypertrophy in removascular hypertensive rats [46] and the hypotensive and anti-platelet effects (inhibits the relaease of platelet-derived tissue factor) in SHR [47]. Effects of Rut to lower systolic blood pressure and reverse mesenteric artery remodeling were found to be related to increased expression of prolylcarboxypeptidase in the circulation and small arteries in renovascular hypertensive rats [48]. However, Rut inhibited platelet aggregation in human platelet-rich plasma by inhibiting TXA_2 _formation, phosphoinositide breakdown and phospholipase C [49-51]. CGRP could work as an endogenous protective substance to counteract endothelial progenitor cells senescence in hypertension and the accelerated endothelial progenitor cells senescence in hypertension is related to the reduction of CGRP while Rut could reverse endothelial progenitor cell senescence along with an elevation in CGRP production in SHR and reverse angiotensin II-induced CGRP mRNA expression in endothelial progenitor cells [52].

Rut solid dispersion significantly increased the blood concentration, accompanied by significant hypotensive effects in SHR in a dose-dependent manner [53]. The 14-N atom of Rut might be the key site for the activity and simple substitute in indole-ring or quinazoline-ring would not enhance the vasodilator effects unless in a proper position and with a proper group [54].

### Effects on Alzheimer's disease

Alzheimer's disease, impairment of memory and cognitive ability caused by the loss of hippocampal and cortical neurons, is related to accumulations of beta-amyloid [55] and disproportionate deficiency of acetylcholine [56]. Treatment for Alzheimer's disease includes transmitter replacement therapies, anti-oxidants, neuronal calcium channel blockers, anti-apoptotic agents, anti-inflammatory agents, estrogens, nerve growth factors and drugs that inhibit secretase activity and prevent or block beta-amyloid formation in the brain [57,58].

DeHE HCl was found to increase the cerebral blood flow in anesthetized cats [59]. In a screening of 29 natural products, *Evodia rutaecarpa *demonstrated a strong inhibitory effect on acetylcholinesterase *in vitro *and an anti-amnesic effect *in vivo*. The active component of *Evodia rutaecarpa *was identified as DeHE HCl [60]. A study suggested that DeHE HCl might be an effective drug not only for the Alzheimer's disease type but also for the vascular type of dementia [61]. Our study reported that DeHE pretreatment attenuated intracerebroventricular administration of beta-amyloid peptide (25-35) and intraperitoneal administration of scopolamine induced amnesia in mice [62]. Furthermore, pre-administration of DeHE *via *vena caudalis for one week effectively improved the Wortmannin and GF-109 203X (WT/GFX) induced spatial memory retention impairment of rats, antagonized tau hyperphosphorylation at multiple Alzheimer's disease site and arrested the overactivation of glycogen synthase kinase-3 induced by WT/GFX [63]. DeHE did not cause any serious adverse effects at the dose levels in the experimental animals [64]. Some novel inhibitors of acetyl- and butyrylcholinesterase derived from DeHE and Rut were also reported [65].

DeHE HCl could provide long-lasting facilitation of synaptic transmission that depended on the activation of both the muscarinic and N-methyl-D-aspartate receptors in the *Cornu Ammonis *area 1 region of rat hippocampal slices on the electrical stimulation evoked field excitatory postsynaptic potentials [66]; however, chronic exposure to DeHE concentration-dependently inhibited glutamate uptake and release in the cultured cerebellar cells [67]. In rat brain slices, DeHE attenuated calyculin A, a protein phosphatase (PP)-2A and PP-1inhibitor, and induced Alzheimer's disease-like tau hyperphosphorylation [68]. *Evodia officinalis *extract demonstrated the most protective effects among 10 kinds of plant extracts against the carboxy-terminal 105 amino acid fragments of amyloid precursor protein induced neurotoxicity [69].

### Themoregulative effects, anti-obese, anti-adipogenic and anti-diabetic effects

Among the Evodia fruit alkaloids(hydroxy-Evo, Evo, Rut and evocarpine), Evo prevented the chlorpromazine induced decrease of body temperature in rats [70]; however, intraperitoneal injection of DeHE or Evo caused a dose-related hypothermia in afebrile rats at 20°C. Moreover, both DeHE and Evo attenuated the febrile response induced by intrahypothalamic injection of exogenous pyrogen in rats [71].

Evo was found to mimic the capsaicin-like anti-obese activities [72]; however, in uncoupling protein-1 (UCP1)-knockout mice, Evo triggered a UCP1-independent mechanism to prevent diet-induced obesity [73]. Furthermore, the anti-adipogenic effects of Evo were not blocked by the specific TRPV1 antagonist capsazepine in 3T3-L1 preadipocytes whereas Evo stimulated the phosphorylation of epidermal growth factor receptor (EGFR), protein kinase C alpha and ERK, all of which were reduced by EGFR inhibitor [74]. Evo inhibited human white preadipocyte differentiation accompanied by up-regulation of both GATA binding protein 2 and 3 mRNA and protein expression [75]. Evo also inhibited the adipocyte differentiation of 3T3-21 and C3H1OT1/2 cells and inhibited the obesity in *db/db *mice. Evo improved the undesirable effects of rosiglitazone, including adipogenesis, body weight gain and hepatotoxicity, while preserving its blood-glucose-lowering effects [76].

Orexin [77] and melanin-concentrating hormone (MCH) [78] regulate food intake, arousal and motivated behavior in lateral hypothalamic area. In fed and in hyperinsulinemic obese mice, insulin signaling led to nuclear exclusion of forkhead transcription factor Foxa2 and reduces expression of MCH and orexin [79]. As constitutive and conditional activation of Foxa2 in the brain increased neuronal MCH and orexin expression, it was suggested that pharmacological inhibition of Foxa2 phosphorylation might improve levels of physical activity, overall health and longevity [80].

Administration of Evo to juvenile rats decreased rate of food intake and body weight increase, reduced orexigenic neuropeptide Y (NPY) and agouti-gene related protein mRNA levels and NPY peptide level but increased the circulating level of leptin [81]. In high-fat-diet-induced (C57BL/6) and leptin-deficient (*ob/ob*) obese mice, Rut ameliorated obesity by inhibiting food intake [82].

Aldose reductase inhibitors are potential drugs for treating diabetic complications [83]. Rhetsinine from *Evodia rutaecarpa *inhibited aldose reductase activity and was considered potentially useful in the treatment of diabetic complications [84].

### GI effects

One of the most important clinic application of Evodia Fructus is treatment of discomfort or chill of stomach.

Water extract of *Evodia rutaecarpa *inhibited the intestinal transit (anti-transit effect) and castor oil-induced diarrhea in mice [85]; however, the water extract of *Evodia rutaecarpa *protected the ethanol-induced rat gastric lesions [86,87]. As mentioned earlier, the protective effects of Rut on acetylsalicylic acid, stress and ethanol-induced gastric mucosa injury were related to stimulation of endogenous CGRP release *via *activation of vanilloid receptor [40,41].

Evo inhibited both gastric emptying and gastrointestinal transit in male rats *via *a mechanism involving cholecystokinin (CCK) release and CCK_1 _receptor activation [88]. DeHE HCl also exhibited anti-transit effect [64].

Anti-emetic effects of the ethanol extracts of WT were demonstrated *via *5-HT and histamine receptors [89].

### Dermatological applications

Among 100 herbal extracts screened for anti-oxidant activity and free radical scavenging activity, *Evodia officinalis *was one of the 14 potential sources of anti-oxidants [90].

*Evodia rutaecarpa*, Evo and Rut inhibited immunoglobulin E (IgE)-antigen complex-induced passive cutaneous anaphylaxis reaction and compound 48/80-induced scratching behaviors in mice. Evo and Rut inhibited IgE-antigen complex-induced TNF-α and IL-4 protein expression in RB2-2H3 cells, suggesting that Evo and Rut could be used for the treatment of atopic dermatitis and rhinitis [91].

Extract of *Evodia officinalis *showed a potent inhibitory effect on ultraviolet B (UVB) induced matrix metalloproteinase (MMP)-1 production in human skin fibroblasts [92]. A defined mixture composed of Rut, DeHE and evodin was shown to inhibit UVB-induced PGE_2 _released by keratinocytes *in vitro *and methyl nicotinate-induced erythema in human skin [93]. Rut also inhibited ultraviolet A (UVA) induced ROS generation and suppressed UVA or H_2_O_2_-induced increase in the expression of MMP-2 and MMP-9 in HaCaT human keratinocytes [94].

### Anti-anoxic effects

Extract of *Evodia rutaecarpa *exerted an antianoxic effect in the KCN-induced anoxia model in mice [95]. Cholinergic mechanism was found to be involved in the antianoxic action of Evo which is an active component of *Evodia rutaecarpa *[96].

### Anti-infectious effects

Anti-infectious, or chemotherapeutic, agents for the treatment of protozoal, helminth, and microbial diseases are not anti-inflammatory agents and different from the pharmacodynamic agents which affected the physiological, biochemical, or immunological function of host. The need to develop new chemotherapeutic agent for the widespred antibiotic-resistant pathogens are very important but less success.

Among 300 herbal extracts screened for the anti-hepatitis B surface antigen capability, *Evodia rutaecarpa *was one of the ten effective herbs [97]. Atanine (3-dimethylallyl-4-methoxy-2-quinolone) was found as an active anthelmintic compound in *Evodia rutaecarpa *[98]. Six quinolone alkaloids (*ie *evocarpine, 1-methyl-2-[(4Z,7Z)-4,7-tridecadienyl]-4(1H)-quinolone, 1-methyl-2-[(6Z,9Z)-6.9-pentadecadienyl]-4(1H)-quinolone, 1-methyl-2-undecyl-4(1H)-quinolone, dihydroevocarpine and 1-methyl-2-pentadecyl-4(1H)-quinolone) isolated from *Evodia rutaecarpa *showed potent anti-*Helicobacter pylori *activity [99]. Two alkyl quinolone compounds, namely 1-methyl-2-[(Z)-8-tridecenyl]-4-(1H)-quinolone and 1-methyl-2-[(Z)-7-tridecenyl]-4-(1H)-quinolone, from *Evodia rutaecarpa *were anti-bacterial agents highly selective *in vitro *against *H. pylori *and almost non-active against other intestinal pathogens [100]. *In vivo *studies on *H. pylori *infected *Mongolian gerbils *demonstrated that alkyl methyl quinolone compounds from *Evodia rutaecarpa *decreased the number of *H. pylori *and inhibited the *H. pylori *respiration [101,102].

Three synthesized 2-alkenyl-4(1H)-quinolone compounds, one of which is found in *Evodia rutaecarpa *demonstrated vasodilating and antibacterial effects [103]. *Evodia rutaecarpa *extract was reported to possess bactericidal activity against gram-positive *cocci*, *P aeruginose *and *C albicans *[104]. Similarly, extracts of *Evodia elleryana *leaves, stem wood, stem bark, root wood, root bark and petrol, dichloromethane, ethyl acetate partition fractions showed a broad spectrum of anti-bacterial activity [105]. Extract of *Evodia elleryana *bark also inhibited *Mycobacterium tuberculosis *[106]. Ethyl acetate extract of *Evodia fatraina *stem bark showed moderate *in vitro *anti-malarial activity against *Plasmodium falciparum *while the ethanol extract exhibited 65% suppression of *Plasmodium berghei *in mice [107].

## Conclusion

Stimulation of CGRP release may partially explain the analgesic, cardiovascular and gastrointestinal protective, anti-obese activities of *Evodia rutaecarpa *and its major bioactive components. Other direct actions by the active components of *Evodia rutaecarpa *on different targets may account for various pharmacological effects of *Evodia rutaecarpa*.

## Abbreviations

5-HT: 5-hydroxytryptamine, serotonin; 5-LO: 5-lipoxygenase; CCK: cholecystokinin; CCR: chemokine receptor; CGRP: calcitonin gene-related peptide; COX: cycloxygenase; DeHE: dehydroevodiamine; EGFR: epidermal growth factor receptor; ERK: extracellular-signal-regulated kinases; Evo: evodiamine; HSV: herpes simplex virus; IgE: immunoglobulin E; IL: interleukin; iNOS: inducible nitric oxide synthase; LIGHT: Homologous to Lymphotoxin, exhibits inducible expression, competes with Herpes Simplex Virus Glycoprotein D for binding to Herpes Virus entry Mediator (HVEM), a receptor on T lymphocytes; LPS: lipopolysaccharide; LT: leukotriene; MCH: melanin-concentrating hormone; MMP: matrix metalloproteinase; NF-kappa B: nuclear factor kappa B; NO: nitric oxide; NOS: nitric oxide synthase; NPY: neuropeptide Y; PG: prostaglandins; PP: protein phosphatase; ROS: reactive oxgen species; Rut: rutaecarpine; SHR: spontaneously hypertensive rats; TNF-α: tumor necrosis factor-alpha; TPH2: tryptophan hydroxylase 2; TRPV1: transient receptor potential channel vanilloid type 1; TX: thromboxanes; UCP1: uncoupling protein-1; UVA: ultraviolet A radiation; UVB: ultraviolet B radiation; WT: Wuzhuyu Tang; WT/GFX: Wortmannin and GF-109 203X

## Competing interests

The authors declare that they have no competing interests.

## Authors' contributions

CFC proposed the review and wrote the manuscript. JFL searched the literature, compiled and reviewed the information and revised the manuscript. WFC reviewed the information on NO, NOS and endotoxaemic rats. YCS reviewed the information on neutrophils and microglial cells. GJW reviewed the information on vascular smooth muscle cell, endothelial cell and electropharmacology. All authors read and approved the final version of the manuscript.

## Supplementary Material

Additional file 1 **Mechanisms of anti-inflammatory relative effects of *Evodia rutaecarpa *and its bioactive components with potential clinic applications**. The known mechanisms for anti-inflammatory effects of *Evodia rutaecarpa *extracts and its bioactive components such as dehydroevodiamine (DeHE), evodiamine (Evo) and rutaecarpine (Rut) are summarized and their potential clinic applications are suggested in this file. Some reported pharmacological effects of Wuzhuyu Tang (composed of Evodia fruit, Ginger, Ginseng, and Jujube) are also listed. Please refer to the text for the detail and references. Click here for file
