# Supplementary material for: Anti-inflammatory and anti-infectious effects of Evodia rutaecarpa (Wuzhuyu) and its major bioactive components
Source: Chin Med. 2011 Feb 14;6:6. doi: 10.1186/1749-8546-6-6 (PMC3046897; doi:10.1186/1749-8546-6-6)
Supplement: Additional file 1 — Mechanisms of anti-inflammatory relative effects of Evodia rutaecarpa and its bioactive components with potential clinic applications. The known mechanisms for anti-inflammatory effects of Evodia rutaecarpa extracts and its bioactive components such as dehydroevodiamine (DeHE), evodiamine (Evo) and rutaecarpine (Rut) are summarized and their potential clinic applications are suggested in this file. Some reported pharmacological effects of Wuzhuyu Tang (composed of Evodia fruit, Ginger, Ginseng, and Jujube) are also listed. Please refer to the text for the detail and references. [file 1749-8546-6-6-S1.DOC]

| **Compounds** | **Mechanism(s) and references** | **Potential clinic application** |
| --- | --- | --- |
| *Evodia rutaecarpa* | Reduces NO over-production in plasma in LPS-induced endotoxaemic rats [13] | Treatment of septic shock |
|  | Inhibits NADPH oxidase activity of neutrophils [14] | Anti-inflammation |
|  | Possesses radical-scavenging properties [14] | Anti-oxidant |
| *Evodia rutaecarpa,* DeHE, Evo, Rut | Inhibit the LPS-induced NO production and iNOS expression in microglial cells [14] | Anti-inflammation |
|  | Stimulate interleukin-1β, interleukin-6, TNF-α, and granulocyte-macrophage colony-stimulating factor secretion [20,21] | Immuno-modulation |
| *Evodia rutaecarpa*, Evo, Rut | Inhibit IgE-antigen complex reaction [91] | Treatment of atopic dermatitis and rhinitis |
| Evo, Rut | Inhibit PGE_2_ synthesis from LPS-treated RAW 264.7 cell [15] | Anti-inflammation |
|  | Decrease ROS production and NADPH oxidase activation [23] | Anti-oxidant |
|  | Stimulate CGRP and substance P synthesis and release [25,28,32,33,35,36] | Analgesics, G-I & C-V protection |
| Evo | Vanilloid receptor agonist [28-30,32,33,35,36] | Analgesics, G-I & C-V protection |
|  | Decreases pyrogen induced febrile response [66,71] | Antipyretics |
|  | Activates ERK and its negative cross-talk with the insulin signaling pathway [68] | Anti-diabetics |
|  | Charybdotoxin-sensitive K^+^ channel activation and nonselective interfering phosphodiesterase to prevent cyclic nucleotide degradation [11 | Treatment of male erectile dysfunction |
|  | Inhibits NO production by interfering with the priming signal initiated by interferon-gamma [12]  Inhibits COX-2 induction and NF-kappa B activation [15]  Dephosphorylation of Akt and p70s6 kinase to regulate the translation process of hypoxia-inducible factor 1 alpha mRNA [17] | Anti-inflammation |
|  | Stimulates the phosphorylation of epidermal growth factor receptor, protein kinase C alpha, and ERK [74]  Inhibits white preadipocyte differentiation by up-regulation of both GATA binding protein 2 and 3 mRNA and protein expression [75]  Improves the effect of insulin [76]  Reduces orexigenic neuropeptide and agouti-gene related protein mRNA [81] | Anti-obesity |
|  | Causes cholecystokinin release and its receptor activation [88] | Inhibits gastric emptying, G-I transit |
| DeHE | 10% endothelium-dependent vasodilatation, α_1_-adrenoceptor and Ca^2+^ channel blockade, K^+^ channel activation [7] | Anti-hypertension |
|  | Inhibits NO production by interfering with priming signal initiated by interferon-gamma and iNOS protein synthesis in RAW264.7 cells [12]  Suppress NF-kappa B activation in the transcriptional level [16] | Anti-inflammation |
|  | Causes hypothermia in afebrile and pyrogen induced febrile in rats [71] | Thermoregulator |
|  | Increases the cerebral blood flow [59]  Acetylcholinesterase inhibitor [60]  Improves brain lesion or ischemia [61]  Attenuates beta-amyloid peptide induced brain damage and antagonizes tau hyperphosphorylation [62,63]  Produces long-lasting facilitation of synaptic transmission [66]  Inhibits glutamate uptake and release [67]  Attenuates calyculin A induced tau hyperphosphorylation [68] | Treatment of Alzheimer's disease |
| Rut | 100% endothelium-dependent vasodilation [6]  Influx of extracellular calcium into endothelial cell [8]  Inhibits the L-type voltage-dependent calcium channel of rat vascular smooth muscle cells and opening of non-voltage-dependent calcium channels in endothelia cells [9,10]  Increases prolylcarboxypeptidase expression in 2K1C hypertensive rats [48]  Hypotensive effect through CGRP stimulation [38] [44-47] [52] | Anti-hypertension |
|  | Cardioprotection through CGRP stimulation [35] [37] | Treatment of antigen induce cardiac anaphylactic injury |
|  | Inhibits hypoxia induced neuronal apoptosis via TRPV1-[Ca^2+^]i-dependent and PI3K/Akt signaling pathway [39] | Neuronal protection |
|  | Gastric mucosa protection by CGRP stimulation [40,41] | G-I protection |
|  | Inhibits platelet aggregation by inhibition of TXA2 formation, phosphoinositide breakdown and phospholipase C [47,49-51] | Anti-platelet effect |
|  | Decreases antigen-induced TNF-α production and is a COX-2 inhibitor [35,18] | Anti-inflammation |
|  | Inhibits expression of orexigenic neuropeptides NPY and agouti-gene related protein [82] | Anti-obesity |
|  | Inhibits UVA-induced ROS generation and expression of MMP-2 and MMP-9 [94] | Sunscreen |
| Rhetsinine | Inhibits aldose reductase activity [84] | Anti-diabetics |
| Goshuyuamide | Inhibits 5-lipoxygenase [15] | Anti-inflammation |
| Wuzhuyu Tang | Stimulates TPH2 promoter, promotes 5-HT synthesis and release [19] | Anti-migraine |
|  | Anti-emetic effect via antagonism to acetylcholine, 5-HT, and histamine receptor [89] | Anti-emetics |
|  | Prevents the chlorpromazine induced decrease of body temperature, and Evo is the active component [70] | Thermoregulators |
